# 2-(4-Bromo­phen­yl)-6-methyl-4*H*-1-benzopyran-4-one (4′-bromo-6-methyl­flavone)

**DOI:** 10.1107/S1600536810010718

**Published:** 2010-03-27

**Authors:** Tomasz Janeczko, Agata Białońska, Edyta Kostrzewa-Susłow

**Affiliations:** a Department of Chemistry, Wrocław University of Environmental and Life Sciences, 25 Norwida, 50-375 Wrocław, Poland; b Faculty of Chemistry, University of Wrocław, 14 F. Joliot-Curie, 50-383 Wrocław, Poland

## Abstract

Planar (r.m.s. deviation from the plane through all non-H atoms = 0.036 Å) mol­ecules of the title compound, C_16_H_11_BrO_2_, form a layered structure stabilized by C—H⋯O hydrogen bonds and π–π stacking inter­actions.

## Related literature

For background information on flavones and their properties, see: Hsiao *et al.* (2007 ▶); Manthey *et al.* (2001 ▶); Middleton *et al.* (2000 ▶). Millot *et al.* (2009 ▶); Moulari *et al.* (2006 ▶); Ren *et al.* (2003 ▶); Moon *et al.* (2007 ▶). For related structures, see: Kumar *et al.* (1998 ▶); Artali *et al.* (2003 ▶); Białońska *et al.* (2007 ▶); Ghalib *et al.* (2010 ▶).
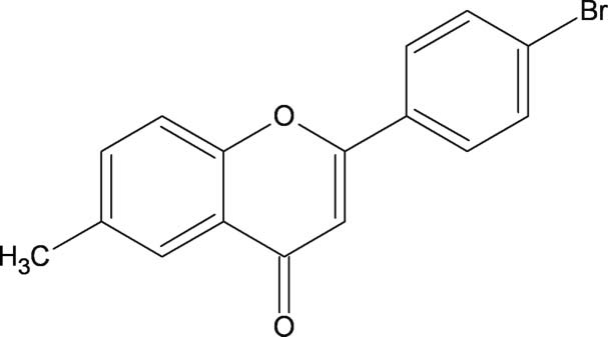


## Experimental

### 

#### Crystal data

C_16_H_11_BrO_2_
*M* *_r_* = 315.16
Monoclinic, 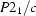

*a* = 13.759 (3) Å
*b* = 6.873 (2) Å
*c* = 13.460 (2) Å
β = 90.25 (3)°
*V* = 1272.8 (5) Å^3^
*Z* = 4
Mo *K*α radiation
μ = 3.22 mm^−1^
*T* = 100 K
0.31 × 0.29 × 0.04 mm

#### Data collection

Kuma KM-4-CCD diffractometer
Absorption correction: analytical [*CrysAlis RED* (Oxford Diffraction, 2009 ▶); analytical numeric absorption correction using a multifaceted crystal model based on expressions derived by Clark & Reid (1995 ▶)] *T*_min_ = 0.474, *T*_max_ = 0.893
25822 measured reflections
5962 independent reflections
3659 reflections with *I* > 2σ(*I*)
*R*_int_ = 0.047

#### Refinement

*R*[*F* ^2^ > 2σ(*F* ^2^)] = 0.028
*wR*(*F* ^2^) = 0.070
*S* = 0.88
5962 reflections
172 parameters
H-atom parameters constrained
Δρ_max_ = 0.69 e Å^−3^
Δρ_min_ = −0.37 e Å^−3^

### 

Data collection: *CrysAlis CCD* (Oxford Diffraction, 2009 ▶); cell refinement: *CrysAlis RED* (Oxford Diffraction, 2009 ▶); data reduction: *CrysAlis RED*; program(s) used to solve structure: *SHELXS97* (Sheldrick, 2008 ▶); program(s) used to refine structure: *SHELXL97* (Sheldrick, 2008 ▶); molecular graphics: *XP* (Bruker, 1999 ▶); software used to prepare material for publication: *SHELXL97*.

## Supplementary Material

Crystal structure: contains datablocks global, I. DOI: 10.1107/S1600536810010718/ds2025sup1.cif

Structure factors: contains datablocks I. DOI: 10.1107/S1600536810010718/ds2025Isup2.hkl

Additional supplementary materials:  crystallographic information; 3D view; checkCIF report

## Figures and Tables

**Table 1:** Hydrogen-bond geometry (Å, °)

*D*—H⋯*A*	*D*—H	H⋯*A*	*D*⋯*A*	*D*—H⋯*A*
C3—H3*A*⋯O4^i^	0.95	2.49	3.2904 (17)	142
C16—H16*A*⋯O4^i^	0.95	2.58	3.4394 (16)	151

Symmetry code: (i) 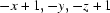.

**Table 2:** π–π inter­actions (Å, °) *Cg*(1) and *Cg*(2) are the centroids of the C5–C10 and C11–C16 rings, respectively.

*Cg*(*I*)	*Cg*(*J*)	*Cg*–*Cg*	Alpha	*CgI*_perp	*CgJ*_Perp	Slippage
*Cg*(1)	*Cg*(2)^i^	3.895	7.13 (3)	3.579 (2)	−3.430 (2)	1.84
*Cg*(1)	*Cg*(2)^ii^	3.843	7.13 (3)	−3.266 (2)	3.438 (2)	1.72

Notes: *Cg*–*Cg* = distance between ring centroids; Alpha = dihedral angle between planes *I* and *J*; *CgI*_Perp = perpendicular distance of *Cg*(*I*) on ring *J*; *CgJ*_Perp = perpendicular distance of *Cg*(*J*) on ring *I*; Slippage = distance between *Cg*(*I*) and perpendicular projection of *Cg*(*J*) on Ring *I*. Symmetry codes: (i) 
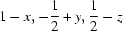
; (ii) 
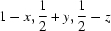
.

**Fig. 1.**
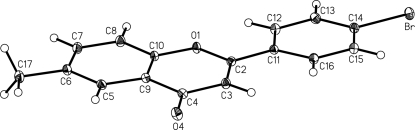
Selected view of 4'-bromo-6-methyloflavone with the numbering scheme employed.

**Fig. 2.**
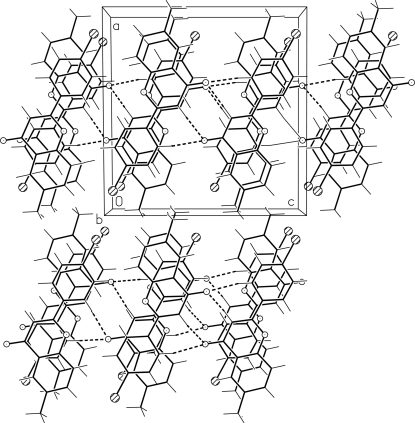
Molecular packing of 4'-bromo-6-methyloflavone.

## supplementary crystallographic information

### Comment

Seeds, fruit skin, bark and flowers of most plants contain significant amount of flavonoids. They have been classified to one subclass of flavonoids according to their chemical structures (Hsiao *et al.*, 2007). Several naturally occurring and synthetic flavones are well know in respect to their anti-oxidant, anti-neoplastic, anti-malarial, anti-inflammatory and insecticidal activity (Manthey *et al.*, 2001; Millot *et al.*, 2009; Moulari *et al.*, 2006). Halogenoflavones have been used as precursors for the synthesis of a variety of bioactive organic compounds including biflavodoids (Ren *et al.*, 2003; Moon *et al.*, 2007). The title compound is a flavone derivative with 4'-bromo and 6-methyl substituents in the biologically active region (Scheme) (Middleton *et al.*, 2000).

Crystal structures of the following related flavones were reported: 7-hydroxyflavone monohydrate (Kumar *et al.*, 1998), 6-(3-hydroxy-3-methylbut-1-ynyl)-flavone and 6-(3-methylbut-3-en-1-ynyl)-flavone (Artali *et al.*, 2003), 2-phenyl-6-hydroxy-4*H*-1-benzopyran-4-one (6-hydroxyflavone) (Białońska *et al.*, 2007), 3,5,4'-trihydroxy-6,7-dimethoxy-flavone (Eupalitin) (Ghalib *et al.*, 2010).

Structure of 2-(4-bromophenyl)-6-methyl-4*H*-1-benzopyran-4-one with the numbering scheme employed is presented in Fig. 1 Molecules of the titled compound form ribbons stabilized by π–π stacking interactions exteded along the [010] direction (Table 2). The neighboring ribbons are linked by C—H···O hydrogen bonds, in which the carbonyl O4 atom is their acceptor (Table 1). The resulting layers perpendicular to the [100] direction (Fig. 2).

### Experimental

The title compound was obtained according to the procedure: A mixture of the para-cresol 1,08 g (10,0 mmol) and 3,4'-dibromopropiophenone 0,59 g (2,0 mmol) in BF3.OEt2 (20 ml) was heated at 60 °C and stirred for 8 h. The products of reaction were extracted from the mixtures with chloroform. Titled product was separated by column chromatography on silica gel with hexane/methyl chloride/acetone (10:1:1 v/v/v) as eluent (Scheme). Crystals suitable for X-ray structure analysis were obtained by slow evaporation from the eluent at room temperature. Structure of the titled product was confirmed by means of the 1H NMR and 13 C NMR spectra. 1H NMR (600 MHz, CDCl3 δ, p.p.m.): 6.81 (s, 1H, H3), 7.48 (d, 1H, J=8.56 Hz, H8), 7.54 (dd, 1H, J=8.56, 2.12 Hz, H7), 7.68 (m, 2H Wh=8.60 Hz, H5' and H7'), 7.80 (m, 2H, Wh=8.60 Hz, H2' and H6'), 8.04 (d, 1H, J=2.12 Hz, H-5). 13 C NMR (150 MHz, CDCl3 δ, p.p.m.): 20.98 (-CH3); 107.55 (C3); 117.83 (C8); 123.56 (C10); 125.13 (C5); 126.25 (C6); 127.71 (C3' i C5'); 130.84 (C1'); 132.35 (C2' i C6'); 135.21 (C7); 135.47 (C4'); 154.48 (C9) 162.23 (C2); 178.48 (C4).

### Refinement

Non-hydrogen atoms were refined with anisotropic displacement parameters. All H atoms were placed at calculated positions and were treated as riding atoms, with C—H distances of 0.95 - 1.00 Å.

### Crystal data

**Table:**

C_16_H_11_BrO_2_	*F*(000) = 632
*M**_r_* = 315.16	*D*_x_ = 1.645 Mg m^−^^3^
Monoclinic, *P*2_1_/*c*	Mo *K*α radiation, λ = 0.71073 Å
Hall symbol: -P 2ybc	Cell parameters from 11464 reflections
*a* = 13.759 (3) Å	θ = 3.0–36.9°
*b* = 6.873 (2) Å	µ = 3.22 mm^−^^1^
*c* = 13.460 (2) Å	*T* = 100 K
β = 90.25 (3)°	Plate, colorless
*V* = 1272.8 (5) Å^3^	0.31 × 0.29 × 0.04 mm
*Z* = 4	

### Data collection

**Table:**

Kuma KM-4-CCD diffractometer	5962 independent reflections
Radiation source: fine-focus sealed tube	3659 reflections with *I* > 2σ(*I*)
graphite	*R*_int_ = 0.047
ω scan	θ_max_ = 36.0°, θ_min_ = 3.0°
Absorption correction: analytical [*CrysAlis RED* (Oxford Diffraction, 2009); analytical numeric absorption correction using a multifaceted crystal model based on expressions derived by Clark & Reid (1995)]	*h* = −22→22
*T*_min_ = 0.474, *T*_max_ = 0.893	*k* = −11→10
25822 measured reflections	*l* = −21→22

### Refinement

**Table:**

Refinement on *F*^2^	Primary atom site location: structure-invariant direct methods
Least-squares matrix: full	Secondary atom site location: difference Fourier map
*R*[*F*^2^ > 2σ(*F*^2^)] = 0.028	Hydrogen site location: inferred from neighbouring sites
*wR*(*F*^2^) = 0.070	H-atom parameters constrained
*S* = 0.88	*w* = 1/[σ^2^(*F*_o_^2^) + (0.0385*P*)^2^] where *P* = (*F*_o_^2^ + 2*F*_c_^2^)/3
5962 reflections	(Δ/σ)_max_ < 0.001
172 parameters	Δρ_max_ = 0.69 e Å^−^^3^
0 restraints	Δρ_min_ = −0.37 e Å^−^^3^

### Special details

**Table:**

Experimental. CrysAlis RED, Oxford Diffraction Ltd., Version 1.171.33.42 (release 29-05-2009 CrysAlis171 .NET) (compiled May 29 2009,17:40:42) Analytical numeric absorption correction using a multifaceted crystal model based on expressions derived by R.C. Clark & J.S. Reid. (Clark, R. C. & Reid, J. S. (1995). Acta Cryst. A51, 887-897)
Geometry. All esds (except the esd in the dihedral angle between two l.s. planes) are estimated using the full covariance matrix. The cell esds are taken into account individually in the estimation of esds in distances, angles and torsion angles; correlations between esds in cell parameters are only used when they are defined by crystal symmetry. An approximate (isotropic) treatment of cell esds is used for estimating esds involving l.s. planes.
Refinement. Refinement of *F*^2^ against ALL reflections. The weighted *R*-factor wR and goodness of fit *S* are based on *F*^2^, conventional *R*-factors *R* are based on *F*, with *F* set to zero for negative *F*^2^. The threshold expression of *F*^2^ > σ(*F*^2^) is used only for calculating *R*-factors(gt) etc. and is not relevant to the choice of reflections for refinement. *R*-factors based on *F*^2^ are statistically about twice as large as those based on *F*, and *R*- factors based on ALL data will be even larger.

### Fractional atomic coordinates and isotropic or equivalent isotropic displacement parameters (Å^2^)

**Table:**

	*x*	*y*	*z*	*U*_iso_*/*U*_eq_	
Br	0.122301 (8)	0.12666 (2)	0.048602 (10)	0.02299 (4)	
O1	0.59232 (6)	0.12638 (15)	0.20324 (6)	0.01526 (15)	
C2	0.51619 (8)	0.12091 (19)	0.26748 (8)	0.01346 (19)	
C3	0.52887 (8)	0.1170 (2)	0.36736 (8)	0.0158 (2)	
H3A	0.4733	0.1138	0.4089	0.019*	
O4	0.63780 (7)	0.11445 (16)	0.50391 (6)	0.02132 (18)	
C4	0.62442 (8)	0.1176 (2)	0.41278 (9)	0.0152 (2)	
C5	0.80289 (8)	0.1197 (2)	0.37337 (9)	0.0158 (2)	
H5A	0.8173	0.1160	0.4424	0.019*	
C6	0.87838 (8)	0.1235 (2)	0.30587 (9)	0.0173 (2)	
C7	0.85504 (9)	0.1311 (2)	0.20364 (9)	0.0188 (2)	
H7A	0.9062	0.1348	0.1565	0.023*	
C8	0.76018 (9)	0.1335 (2)	0.17014 (9)	0.0179 (2)	
H8A	0.7459	0.1396	0.1011	0.021*	
C9	0.70523 (8)	0.1212 (2)	0.34182 (8)	0.01366 (19)	
C10	0.68548 (8)	0.1267 (2)	0.24016 (9)	0.01469 (19)	
C11	0.42181 (8)	0.12034 (19)	0.21497 (8)	0.01378 (19)	
C12	0.41819 (9)	0.1370 (2)	0.11092 (9)	0.0170 (2)	
H12A	0.4768	0.1477	0.0743	0.020*	
C13	0.32929 (9)	0.1380 (2)	0.06124 (9)	0.0181 (2)	
H13A	0.3269	0.1486	−0.0091	0.022*	
C14	0.24418 (8)	0.1234 (2)	0.11563 (9)	0.0169 (2)	
C15	0.24576 (9)	0.1070 (2)	0.21903 (10)	0.0184 (2)	
H15A	0.1869	0.0980	0.2553	0.022*	
C16	0.33448 (9)	0.1041 (2)	0.26785 (9)	0.0166 (2)	
H16A	0.3363	0.0910	0.3381	0.020*	
C17	0.98418 (9)	0.1213 (2)	0.33957 (10)	0.0217 (2)	
H17A	0.9871	0.1160	0.4123	0.033*	
H17B	1.0167	0.2396	0.3163	0.033*	
H17C	1.0168	0.0069	0.3118	0.033*	

### Atomic displacement parameters (Å^2^)

**Table:**

	*U*^11^	*U*^22^	*U*^33^	*U*^12^	*U*^13^	*U*^23^
Br	0.01513 (6)	0.02745 (7)	0.02633 (7)	0.00270 (6)	−0.00771 (4)	−0.00266 (6)
O1	0.0108 (3)	0.0227 (4)	0.0123 (3)	−0.0008 (4)	−0.0008 (3)	0.0003 (4)
C2	0.0130 (4)	0.0128 (5)	0.0145 (5)	−0.0002 (5)	0.0009 (4)	0.0007 (5)
C3	0.0135 (4)	0.0200 (5)	0.0139 (5)	−0.0007 (5)	0.0013 (4)	0.0001 (5)
O4	0.0200 (4)	0.0308 (5)	0.0132 (4)	−0.0045 (4)	−0.0010 (3)	0.0008 (4)
C4	0.0155 (5)	0.0155 (5)	0.0145 (5)	−0.0015 (5)	−0.0006 (4)	0.0003 (5)
C5	0.0146 (5)	0.0159 (5)	0.0169 (5)	−0.0008 (5)	−0.0031 (4)	−0.0006 (5)
C6	0.0128 (4)	0.0177 (5)	0.0215 (5)	−0.0009 (5)	−0.0020 (4)	−0.0009 (5)
C7	0.0137 (5)	0.0232 (6)	0.0195 (5)	−0.0004 (5)	0.0019 (4)	−0.0014 (6)
C8	0.0147 (5)	0.0239 (6)	0.0150 (5)	−0.0004 (5)	0.0004 (4)	−0.0012 (5)
C9	0.0124 (4)	0.0143 (5)	0.0144 (5)	−0.0010 (5)	−0.0005 (4)	0.0002 (5)
C10	0.0115 (4)	0.0157 (5)	0.0168 (5)	−0.0008 (5)	−0.0012 (4)	−0.0007 (5)
C11	0.0122 (4)	0.0138 (5)	0.0154 (5)	−0.0001 (5)	−0.0015 (4)	−0.0002 (5)
C12	0.0149 (5)	0.0191 (6)	0.0171 (5)	−0.0007 (5)	−0.0005 (4)	0.0017 (5)
C13	0.0178 (5)	0.0195 (6)	0.0170 (5)	−0.0008 (5)	−0.0031 (4)	0.0008 (5)
C14	0.0144 (5)	0.0160 (5)	0.0203 (5)	0.0017 (5)	−0.0046 (4)	−0.0008 (5)
C15	0.0139 (5)	0.0196 (6)	0.0216 (6)	0.0003 (5)	−0.0001 (4)	−0.0012 (5)
C16	0.0138 (5)	0.0198 (6)	0.0163 (5)	−0.0001 (5)	0.0000 (4)	−0.0002 (5)
C17	0.0140 (5)	0.0270 (6)	0.0240 (6)	0.0002 (6)	−0.0036 (4)	−0.0022 (6)

### Geometric parameters (Å, °)

**Table:**

Br—C14	1.9008 (13)	C8—C10	1.3983 (16)
O1—C2	1.3617 (14)	C8—H8A	0.9500
O1—C10	1.3727 (14)	C9—C10	1.3944 (16)
C2—C3	1.3551 (16)	C11—C16	1.4036 (17)
C2—C11	1.4757 (16)	C11—C12	1.4059 (16)
C3—C4	1.4475 (17)	C12—C13	1.3914 (17)
C3—H3A	0.9500	C12—H12A	0.9500
O4—C4	1.2397 (15)	C13—C14	1.3875 (17)
C4—C9	1.4692 (16)	C13—H13A	0.9500
C5—C6	1.3832 (17)	C14—C15	1.3964 (18)
C5—C9	1.4075 (16)	C15—C16	1.3839 (17)
C5—H5A	0.9500	C15—H15A	0.9500
C6—C7	1.4126 (18)	C16—H16A	0.9500
C6—C17	1.5228 (17)	C17—H17A	0.9800
C7—C8	1.3792 (17)	C17—H17B	0.9800
C7—H7A	0.9500	C17—H17C	0.9800
			
C2—O1—C10	119.33 (9)	O1—C10—C8	116.36 (10)
C3—C2—O1	122.30 (10)	C9—C10—C8	121.44 (10)
C3—C2—C11	125.75 (11)	C16—C11—C12	119.01 (11)
O1—C2—C11	111.95 (9)	C16—C11—C2	120.72 (10)
C2—C3—C4	122.12 (11)	C12—C11—C2	120.27 (10)
C2—C3—H3A	118.9	C13—C12—C11	120.42 (11)
C4—C3—H3A	118.9	C13—C12—H12A	119.8
O4—C4—C3	123.27 (11)	C11—C12—H12A	119.8
O4—C4—C9	122.28 (11)	C14—C13—C12	119.23 (11)
C3—C4—C9	114.45 (10)	C14—C13—H13A	120.4
C6—C5—C9	121.35 (11)	C12—C13—H13A	120.4
C6—C5—H5A	119.3	C13—C14—C15	121.48 (11)
C9—C5—H5A	119.3	C13—C14—Br	119.58 (9)
C5—C6—C7	118.19 (11)	C15—C14—Br	118.94 (9)
C5—C6—C17	121.59 (11)	C16—C15—C14	118.97 (12)
C7—C6—C17	120.23 (11)	C16—C15—H15A	120.5
C8—C7—C6	121.99 (11)	C14—C15—H15A	120.5
C8—C7—H7A	119.0	C15—C16—C11	120.88 (12)
C6—C7—H7A	119.0	C15—C16—H16A	119.6
C7—C8—C10	118.46 (11)	C11—C16—H16A	119.6
C7—C8—H8A	120.8	C6—C17—H17A	109.5
C10—C8—H8A	120.8	C6—C17—H17B	109.5
C10—C9—C5	118.56 (10)	H17A—C17—H17B	109.5
C10—C9—C4	119.58 (10)	C6—C17—H17C	109.5
C5—C9—C4	121.87 (10)	H17A—C17—H17C	109.5
O1—C10—C9	122.20 (10)	H17B—C17—H17C	109.5
			
C10—O1—C2—C3	0.45 (19)	C4—C9—C10—O1	−0.9 (2)
C10—O1—C2—C11	−179.69 (13)	C5—C9—C10—C8	−0.9 (2)
O1—C2—C3—C4	−0.3 (2)	C4—C9—C10—C8	178.91 (13)
C11—C2—C3—C4	179.90 (13)	C7—C8—C10—O1	−179.04 (14)
C2—C3—C4—O4	179.89 (13)	C7—C8—C10—C9	1.2 (2)
C2—C3—C4—C9	−0.5 (2)	C3—C2—C11—C16	−3.7 (2)
C9—C5—C6—C7	0.7 (2)	O1—C2—C11—C16	176.44 (12)
C9—C5—C6—C17	−179.82 (14)	C3—C2—C11—C12	175.99 (13)
C5—C6—C7—C8	−0.4 (2)	O1—C2—C11—C12	−3.86 (18)
C17—C6—C7—C8	−179.95 (14)	C16—C11—C12—C13	0.2 (2)
C6—C7—C8—C10	−0.5 (2)	C2—C11—C12—C13	−179.51 (13)
C6—C5—C9—C10	0.0 (2)	C11—C12—C13—C14	0.3 (2)
C6—C5—C9—C4	−179.84 (13)	C12—C13—C14—C15	−0.2 (2)
O4—C4—C9—C10	−179.34 (13)	C12—C13—C14—Br	179.51 (11)
C3—C4—C9—C10	1.0 (2)	C13—C14—C15—C16	−0.4 (2)
O4—C4—C9—C5	0.5 (2)	Br—C14—C15—C16	179.84 (11)
C3—C4—C9—C5	−179.17 (13)	C14—C15—C16—C11	1.0 (2)
C2—O1—C10—C9	0.1 (2)	C12—C11—C16—C15	−0.8 (2)
C2—O1—C10—C8	−179.67 (12)	C2—C11—C16—C15	178.85 (14)
C5—C9—C10—O1	179.28 (13)		

### Hydrogen-bond geometry (Å, °)

**Table:**

*D*—H···*A*	*D*—H	H···*A*	*D*···*A*	*D*—H···*A*
C3—H3A···O4^i^	0.95	2.49	3.2904 (17)	142
C16—H16A···O4^i^	0.95	2.58	3.4394 (16)	151

Symmetry codes: (i) −*x*+1, −*y*, −*z*+1.

### Table 2 π–π interactions (Å, °).

*Cg*(1) and *Cg*(2) are the centroids of the C5–C10 and C11–C16 rings, respectively.

**Table:**

*Cg*(*I*)	*Cg*(*J*)	*Cg*–*Cg*	Alpha	*Cg**I*_perp	*Cg**J*_Perp	Slippage
*Cg*(1)	*Cg*(2)^i^	3.895	7.13 (3)	3.579 (2)	-3.430 (2)	1.84
*Cg*(1)	*Cg*(2)^ii^	3.843	7.13 (3)	-3.266 (2)	3.438 (2)	1.72

Notes: *Cg*–*Cg*    = distance between ring centroids; Alpha    = dihedral angle between planes *I* and *J*; *Cg**I*_Perp = perpendicular distance of *Cg*(*I*) on ring *J*; *Cg**J*_Perp = perpendicular distance of *Cg*(*J*) on ring *I*; Slippage = distance between *Cg*(*I*) and perpendicular projection of *Cg*(*J*) on Ring *I*. Symmetry codes: (i) 1-x, -0.5+y, 0.5-z, (ii) 1-x, 0.5+y, 0.5-z.
